# Response gene to complement-32 enhances metastatic phenotype by mediating transforming growth factor beta-induced epithelial-mesenchymal transition in human pancreatic cancer cell line BxPC-3

**DOI:** 10.1186/1756-9966-31-29

**Published:** 2012-03-29

**Authors:** Liang Zhu, Hua Qin, Pei-Yuan Li, Sheng-Nan Xu, Hui-Fang Pang, Hui-Zhen Zhao, De-Min Li, Qiu Zhao

**Affiliations:** 1 Department of Gastroenterology, Tongji Hospital, Tongji Medical College, Huazhong University of Science and Technology, Wuhan 430030, China; 2 Department of Gastroenterology, The First Affiliated Hospital of Nanchang University, Nanchang 330006, China

**Keywords:** Response gene to complement-32, Pancreatic cancer, Transforming growth factor -β, Epithelial-mesenchymal transition, Migration

## Abstract

**Background:**

Response gene to complement-32 (RGC-32) is comprehensively expressed in many kinds of tissues and has been reported to be expressed abnormally in different kinds of human tumors. However, the role of RGC-32 in cancer remains controversial and no reports have described the effect of RGC-32 in pancreatic cancer. The present study investigated the expression of RGC-32 in pancreatic cancer tissues and explored the role of RGC-32 in transforming growth factor-beta (TGF-β)-induced epithelial-mesenchymal transition (EMT) in human pancreatic cancer cell line BxPC-3.

**Methods:**

Immunohistochemical staining of RGC-32 and E-cadherin was performed on specimens from 42 patients with pancreatic cancer, 12 with chronic pancreatitis and 8 with normal pancreas. To evaluate the role of RGC-32 in TGF-β-induced EMT in pancreatic cancer cells, BxPC-3 cells were treated with TGF-β1, and RGC-32 siRNA silencing and gene overexpression were performed as well. The mRNA expression and protein expression of RGC-32 and EMT markers such E-cadherin and vimentin were determined by quantitative reverse transcription-PCR (qRT-PCR) and western blot respectively. Finally, migration ability of BxPC-3 cells treated with TGF-β and RGC-32 siRNA transfection was examined by transwell cell migration assay.

**Results:**

We found stronger expression of RGC-32 and higher abnormal expression rate of E-cadherin in pancreatic cancer tissues than those in chronic pancreatitis tissues and normal pancreatic tissues. Immunohistochemical analysis revealed that both RGC-32 positive expression and E-cadherin abnormal expression in pancreatic cancer were correlated with lymph node metastasis and TNM staging. In addition, a significant and positive correlation was found between positive expression of RGC-32 and abnormal expression of E-cadherin. Furthermore, in vitro, we found sustained TGF-β stimuli induced EMT and up-regulated RGC-32 expression in BxPC-3 cells. By means of siRNA silencing and gene overexpression, we further demonstrated that RGC-32 mediated TGF-β-induced EMT and migration in BxPC-3 cells.

**Conclusions:**

The results above indicated that RGC-32 might be a novel metastasis promoting gene in pancreatic cancer and it enhances metastatic phenotype by mediating TGF-β-induced EMT in human pancreatic cancer cell line BxPC-3.

## Background

Pancreatic cancer is one of the most lethal human cancers due to its high metastatic potential, late manifestation of symptoms and strong chemoresistance [1]. Although more and more therapies including surgical resection, chemotherapy and radiotherapy have been used in recent years, patients' overall 5-year survival rate is still less than 5% [2]. Critical progress needs to be made with regard to the metastasis mechanism of pancreatic cancer for early diagnosis and treatment of this deadly disease.

Accumulating evidences have indicated that epithelial-mesenchymal transition (EMT), which was originally found in embryogenesis, contributes to tumor invasion, metastatic dissemination and acquisition of therapeutic resistance [3]. During the process of EMT, epithelial cells change from their epithelial characteristics including cell-cell adhesion, apical-basal polarity and lack of motility to mesenchymal features, such as invasiveness, motility and high resistance to cell death [3]. Besides, a series of molecular events occur including down-regulation of epithelial markers such as E-cadherin and up-regulation of mesenchymal markers such as N-cadherin and vimentin [4].

Transforming growth factor-beta (TGF-β) is a ubiquitously multifunctional cytokine which controls lots of biological events such as development, differentiation and survival of essentially all cell types and tissues [5]. Recently, increasing attention has been paid to its role in the regulation of tumor development and progression. TGF-β is known to play a dual role in tumorigenesis. TGF-β exerts antiproliferative effects in an early phase of tumorigenesis while contributes to tumor progression with aberrations in TGF-β signaling system in later stages of tumorigenesis [5]. TGF-β overexpression has been found in most pancreatic cancer and clinicopathological analysis showed that TGF-β expression was significantly correlated with lymph node metastasis and the depth of invasion [5]. TGF-β and its downstream signaling molecules have been shown to play a critical role in EMT of pancreatic cancer [6-9]. However, the mechanism by which TGF-β induces EMT has not been clear yet.

Response gene to complement (RGC)-32 was first cloned by Badea et al. in 1998 and was comprehensively expressed in many kinds of tissues such as placenta, kidney, pancreas, liver, heart, brain, etc. [10,11]. It has been reported that RGC-32 plays an important role in cell proliferation and differentiation [11,12]. However, the role of RGC-32 in cancer remains controversial. RGC-32 expression has been found to be up-regulated in tumors such as colon, breast and prostate cancer but down-regulated in advanced stages of primary astrocytomas [13,14]. Similarly, studies on RGC-32 mRNA expression in various metastatic cancers have also yielded different results [15,16].These studies suggested that RGC-32 plays a complex role in cancer and the effect of RGC-32 may vary among cancers of different organs or tissues. Until now, to our knowledge, no reports have described the role of RGC-32 in pancreatic cancer. In the present study, we found for the first time that the expression of RGC-32 was up-regulated in pancreatic cancer and was correlated with lymph node metastasis and TNM staging. In addition, a significant and positive correlation was found between positive expression of RGC-32 and abnormal expression of E-cadherin. Furthermore, we demonstrated that RGC-32, as a downstream target of TGF-β, played an important role in inducing EMT as well as promoting cell migration in human pancreatic cancer cell line BxPC-3. The results above implicated that RGC-32 might serve as a novel metastasis promoting factor and promote tumor metastasis by mediating TGF-β-induced EMT.

## Materials and methods

### Tissue samples

The study was approved by the Ethics Committee of Tongji Hospital of Tongji medical college, and informed consent was obtained from each patient. Tumor samples were obtained from 42 patients with pancreatic cancer who had underwent surgery at Tongji Hospital of Tongji Medical College, Wuhan, China during 2005 and 2010. Another 12 chronic pancreatitis tissues and 8 normal pancreatic tissues serving for control were obtained from the same hospital. None of these patients received preoperative treatment, such as chemotherapy or radiotherapy. All of the tumors were confirmed to be pancreatic cancer by clinicopathological examinations. All the cases were classified according to the latest AJCC cancer staging manual [17].

### Immunohistochemistry

All the resected specimens were fixed in 10% buffered formalin and embedded in paraffin. Sections were prepared, and deparaffinized through graded alcohol and xylene, and then washed three times with cold 0.01 mol/L phosphate-buffered saline (PBS). Afterwards, endogenous peroxidase was blocked with 3% hydrogen peroxide in methanol for 20 min. The sections were washed again in PBS three times. Antigen retrieval was accomplished by boiling the slides in the autoclave for 10 min in 10 mmol/L sodium citrate. After treatment with 10% bovine serum, the sections were incubated overnight at 4°C with rabbit polyclonal antibody against RGC-32 (Santa Cruz Biotechnology, USA, diluted 1:50) and E-cadherin (ProteinTech Group, Inc., USA, diluted 1:100), followed by incubation with biotinylated goat anti-rabbit IgG and the streptavidin-biotin peroxidase reagent (SP kit, ZhongShan goldenbridge biotechnology CO. LTD, China). For the negative control, the immunostaining processes were performed by replacing the primary antibody with PBS. Finally, the reaction was visualized with a chromogen, diaminobenzidine in 3% hydrogen peroxidase. Sections were then counterstained with hematoxylin, dehydrated and mounted. Slides were evaluated by two independent pathologists who were blinded to the clinicopathological details.

The intensity of RGC-32 staining was graded as previously described [18]: negative (-), slight positive (+), positive (++), and highly positive (+++). The expression of E-cadherin was judged as two categories, normal and abnormal according to the method previously described [19]: the staining pattern was classified into four groups. Only a membranous pattern, which stained as strongly as normal epithelial cells, was judged as normal. In contrast, absent pattern (loss of staining), cytoplasmic pattern (cytoplasmic staining with loss of membranous expression) and heterogeneous pattern (cytoplasmic staining with preservation of membranous expression) were considered abnormal.

### Cell culture and morphologic analysis

Human pancreatic cancer cell line BxPC-3 was purchased from American Type Culture Collection (ATCC) and cultured in RPMI-1640 medium containing 10% fetal bovine serum (FBS) at 37°C in a 95% O_2 _and 5% CO_2 _incubator. BxPC-3 cells were grown to about 60% confluency in RPMI-1640+ 10% FBS and were then serum-deprived overnight in RPMI-1640 medium. Cells were then treated with 10 ng/ml TGF-β1 (Peprotech Inc., USA.) for 72 h. The morphology of cells was visualized with a phase contrast microscope (×200, Nikon, Japan) and imaged with digital photography.

### Construction of RGC-32 expression plasmid and RGC-32 short interfering RNA (siRNA)

RGC-32 cDNA was amplified from mRNA extracted from BxPC-3 cells and then cloned into pcDNA3.1/*myc*-His C expression vector(Invitrogen, USA)between Hind *III *and BamH *I *restriction sites. The cloned cDNA was verified by sequencing. siRNA targeting human RGC-32 (5' CAGAUUCACUUUAUAGGAA 3' and 5' UUCCUAUAAAGUGAAUCUG 3' duplex) was synthesized by Ribobio Co. (Guang Zhou, China). A scrambled duplex siRNA was used as the negative control.

### Transient transfection of RGC-32 expression plasmid

BxPC-3 cells were plated at 3 × 10^5^/well in 6-well plates and incubated until they reached 95% confluency. Cells were then transiently transfected with lipofectamine 2000 (Invitrogen, USA) according to the manufacturer's recommendations. 4.0 μg of plasmid DNA and 10 μl of lipofectamine 2000 were diluted separately in Opti-MEM I medium (Gibco, USA) and incubated for 5 min. They were then combined and incubated for 30 min at room temperature. The complexes were added to each well and mixed gently, followed by incubation at 37°C. 5 h later, the medium was replaced with RPMI-1640 medium containing 10% fetal bovine serum. Cells were then incubated for 48 h and 72 h respectively for RNA isolation and protein exaction.

### siRNA transfection

BxPC-3 cells were plated at 1 × 10^5^/well in 6-well plates and incubated until they reached 50% confluency. Cells were transfected with RGC-32 siRNA or the negative control siRNA at a final concentration of 50 nM with lipofectamine 2000 according to the manufacturer's instructions. 6 h after initiation of transfection, cells were starved in serum-free RPMI-1640 for another 6 h, followed by treatment with or without 10 ng/ml TGF-β1 for 72 h.

### RNA isolation and quantitative reverse transcription-PCR (qRT-PCR)

Total RNA was isolated from BxPC-3 cells by TRIzol reagent (Invitrogen, USA) according to the manufacturer's instructions and was resuspended in nuclease-free water. 2 μg of total RNA was added to 25 μl of reverse transcription reaction mixture containing 5 μl of 5 × RT Reaction Buffer, 3 μl of dNTPs (10 mmol/L), 1 μl of Oligo (dT), 1 μl of M-MLV (Promega, USA), 1 μl of Rnasin (Fermentas, USA) and indicated amount of DEPC water. The reverse transcription reaction was processed at 70°C for 5 min, 37°C for 1 h and 85°C for 10 min. qPCR was performed with StepOne Real-time PCR systems (ABI, USA) in a reaction volume of 20 μl containing 2 μl of cDNA, 0.8 μl of forward primer (10 nM), 0.8 μl of reverse primer (10 nM), 10 μl of SYBR Green Realtime PCR Master Mix (Toyobo, Japan) and 6.4 μl of ddH_2_O. The qPCR was processed at 95°C for 60 s, followed by 40 cycles of 95°C for 15 s and 60°C for 30 s (data collection). All the qPCR reactions were performed in triplicate. The analysis of qPCR was carried out using the 2^-ΔΔCt ^method. β-actin was taken as the internal control. The nucleotide sequences of the primers were listed in Table 1. All the primers were synthesized by Shanghai Sangon Biological Engineering & Technology and Service Co. Ltd, China.

**Table 1:** PCR primers used in the experiments

Target mRNA	Primer sequences 5'-3'	Product Size (bp)	Gene Bank Accession No
RGC-32 sense	TGCCAGAGGGGACAAAGAC	127	NM_014059.2
RGC-32 antisense	GCAAGCAGGTAAACAAAGTCAG		
E-cadherin sense	ACAGCCCCGCCTTATGATTCTC	140	NM_004360.3
E-cadherin antisense	AAGCGATTGCCCCATTCGTT		
vimentin sense	CCTTGAACGCAAAGTGGAATC	106	NM_003380.3
vimenin antisense	GACATGCTGTTCCTGAATCTGAG		
β-actin sense	GTTGCGTTACACCCTTTCTTG	157	NM_001101.3
β-actin antisense	GACTGCTGTCACCTTCACCGT		

### Western blot

Total protein extraction from BxPC-3 cells and western blot analysis was performed following the protocol as described previously [20]. Briefly, 80 μg of cell protein was eletrophoresed on a 12% SDS/polyacrylamide gel in Tris-glycin buffer and transferred to nitrocellulose membranes. The nitrocellulose membranes were then blocked at room temperature for 2 h in blocking buffer (5% skim milk in TBST) and incubated with RGC-32 antibody (diluted 1:200), E-cadherin antibody (diluted 1:400) and vimentin antibody (ProteinTech Group, Inc., USA, diluted 1:1000) respectively overnight at 4°C with β-actin antibody (ProteinTech Group, Inc., USA, diluted 1:1000) as control. Washed thrice with TBST, nitrocellulose membranes were incubated in HRP-conjugated goat anti-rabbit secondary antibody (Boster, China, diluted 1:3000) for 1 h at room temperature. Extensive washed with TBST, the complex was detected by Super Signal West Pico Chemiluminescent Substrate (Thermo Fisher Scientific Inc, USA) according to the manufacturer's instructions. Blot was scanned and densitometric analysis was done by Image J software (National Institutes of Health, USA).

### Transwell cell migration assay

BxPC-3 cells were transfected with RGC-32 siRNA or the negative control siRNA and treated with 10 ng/ml TGF-β1 or not as described above. 24 h later, the cells were trypsinized, adjusted to 1 × 10^6^/ml in RPMI-1640 medium, and 200 μl of the resuspended cell solution was added to the top chamber of 24-well transwell plates. The bottom chamber was filled with 600 μl of RPMI-1640 medium containing 10% FBS. The transwell plate was assembled and incubated at 37°C in a 5% CO_2 _incubator. 24 h later, the top chamber was removed, washed with PBS, and fixed with 40 ml/l paraformaldehyde for 20 min. Unmigrated cells staying at the upper layer of the microporous membrane were gently scraped with a wet cotton swab and the migrated cells at the lower layer were stained by 0.1% of crystal violet for 10 min. The top chamber was then washed with PBS to remove excess stain and dried. The stained migrated cells were visualized with the phase contrast microscope. The average number of migrated cells per field was quantified under high power (×200).

### Statistical analysis

Data were presented as mean ± standard deviation (SD). Experiments were repeated at least three times. SPSS 17.0 software (IBM, USA) was used for data analysis. Group differences were analyzed by Student t test, analysis of variance (ANOVA), χ^2 ^test or Fisher exact test according to the data type. Spearman rank correlation analysis was used to examine the correlation between RGC-32 positive expression and E-cadherin abnormal expression in pancreatic cancer tissues. P < 0.05 was considered statistically significant.

## Results

### The expression of RGC-32 and E-cadherin in normal pancreas, chronic pancreatitis and pancreatic cancer tissues and the relationships with clinicopathological features

**Figure 1:**
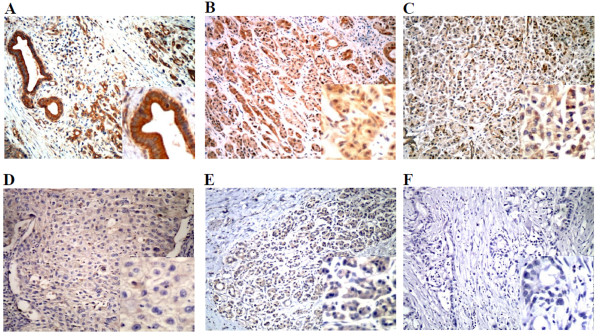
**Representative immunohistochemical staining for RGC-32(A-C) and E-cadherin (D-F) in pancreatic cancer, chronic pancreatitis and normal pancreas tissues (original magnification × 200)**. (A) RGC-32 highly positive staining in pancreatic cancer tissues (B) RGC-32 positive staining in chronic pancreatitis tissues (C) RGC-32 slightly positive staining in normal pancreas tissues (D) normal membranous E-cadherin staining (membranous pattern) in pancreatic cancer tissues (E) cytoplasmic staining with loss of membranous expression (cytoplasmic pattern) in pancreatic cancer tissues (F) loss of E-cadherin staining (absent pattern) in pancreatic cancer tissues.

Immunohistochemical staining revealed that RGC-32 was expressed in pancreatic cancer as well as chronic pancreatitis and normal pancreas. RGC-32 staining was predominantly observed in the cytoplasm of pancreatic acinar cells (Figure 1A-C). Both the positive expression rate and staining intensity of RGC-32 in pancreatic cancer tissues were significantly higher than those in normal pancreatic tissues and pancreatitis tissues, but no significant differences were found between normal pancreatic tissues and pancreatitis tissues (Table 2).

**Table 2:** Expression of RGC-32 and E-cadherin in normal pancreas, chronic pancreatitis and pancreatic cancer tissues

Tissue	RGC-32 staining intensity						E-cadherin		
	
	-	+	++	+++	Positive/total	P-value	normal	abnormal	P-value
Normal pancreas	5	3	0	0	3/8	1.000^a^	8	0	1.000^a^
Chronic pancreatitis	7	3	2	0	5/12	0.028^b^	11	1	0.004^b^
Pancreatic cancer	9	5	12	16	33/42	0.030^c^	19	23	0.005^c^

a, normal pancreas compared with chronic pancreatitis

b, chronic pancreatitis compared with pancreatic cancer

c, pancreatic cancer compared with normal pancreas

The positive staining for E-cadherin was mainly observed in the cytomembrane of normal pancreatic acinar cells (normal expression), but might be found in the cytoplasm and/or become weaker in pancreatic cancer cells (abnormal expression) (Figure 1D-F). The abnormal expression rate of E-cadherin was significantly increased in pancreatic cancer tissues compared with normal pancreas and chronic pancreatitis tissues, but no significant differences were found between normal pancreatic tissues and pancreatitis tissues (Table 2).

The relationships between immunostaining and clinicopathological characteristics of all 42 pancreatic cancer patients were shown in Table 3. Age and gender showed no correlation with either RGC-32 or E-cadherin (P > 0.05). Both lymph node metastasis and TNM staging were significantly correlated with RGC-32 and E-cadherin (P < 0.05). The positive expression rate of RGC-32 and the abnormal expression rate of E-cadherin were found to be increased in tumors with a less advanced pathological stage and higher TNM classification. Tumor differentiation was also correlated with abnormal expression rate of E-cadherin (P < 0.05) but not with the expression of RGC-32 (P > 0.05). The abnormal E-cadherin expression rate was higher in poorly-differentiated-type tumors than in well-differentiated-type counterparts.

**Table 3:** Correlation between clinicopathological findings and immunochemical staining

	cases	RGC-32 positive			Abnormal E-cadherin		
	
		n	%	P-value	n	%	P-value
Age				0.831			0.990
< 45	7	5	71.4		4	57.1	
45-59	22	18	81.8		12	54.5	
> = 60	13	10	76.9		7	53.8	
Gender				1.000			1.000
Male	21	17	81.0		11	52.4	
Female	21	16	76.2		12	57.1	
Differentiation				0.629			0.024
Well	16	12	75.0		5	31.3	
Moderately	11	8	72.7		6	54.5	
Poorly	15	13	86.7		12	80.0	
Lymph node metastasis				0.016			0.004
Negative	16	9	56.3		4	25.0	
Positive	26	24	92.3		19	73.1	
TNM staging				0.025			0.004
I-II	18	11	61.1		5	27.8	
III-IV	24	22	91.7		18	75.0	

Furthermore, a significant and positive correlation was found between positive expression of RGC-32 and abnormal expression of E-cadherin (R = 0.458, P < 0.01, Table 4).

**Table 4:** Correlation between RGC-32 expression and E-cadherin expression in pancreatic cancer tissues

		E-cadherin			
		
		abnormal	normal	R-value	P-value
RGC-32	+	22	11	0.458	0.002
	-	1	8		

## TGF-β induces EMT and enhances RGC-32 expression in BxPC-3 cells

**Figure 2:**
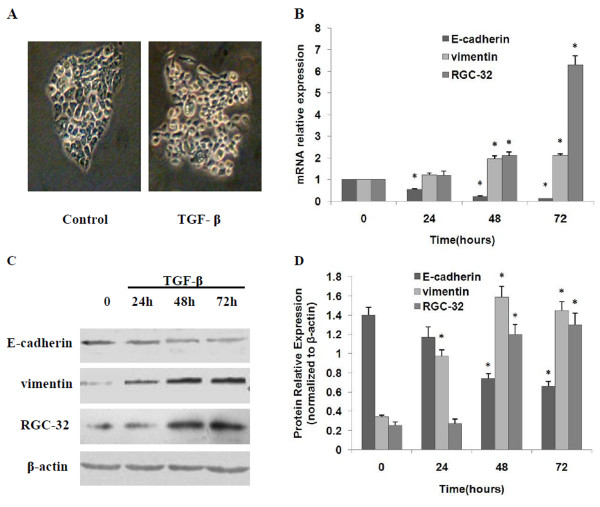
**TGF-β induces EMT and enhances RGC-32 expression in BxPC-3 cells**. BxPC-3 cells were cultured and treated with 10 ng/ml of TGF-β1 for 24 h, 48 h and 72 h, respectively. The morphology of cells at 72 h of TGF-β1 treatment was visualized with a phase contrast microscope (original magnification × 200, Nikon). (A) mRNA expression of E-cadherin, vimentin and RGC-32 was quantified by qRT-PCR with β-actin as an internal control. (B) Protein expression of E-cadherin, vimentin and RGC-32 was detected by western blot, (C) and normalized by β-actin (D). *P < 0.05 compared with the control group (0 h).

TGF-β1 (10 ng/ml) treatment of pancreatic cancer cell line BxPC-3 for 72 h caused remarkable changes in cell morphology from a more epithelial-like appearance to a mesenchymal-like spindle-cell shape and increased intercellular separation (Figure 2A). Furthermore, qRT-PCR showed that E-cadherin mRNA expression was gradually decreased with significant down-regulation at 48 h of TGF-β1 treatment and remained descending until 72 h, the longest time tested, while vimentin mRNA expression was up-regulated significantly as early as 24 h of TGF-β1 treatment and remained increasing until 72 h (Figure 2B). Western blot detecting E-cadherin and vimentin protein expression showed similar results (Figure 2C and 2D). Taken together, we confirmed that sustained TGF-β1 stimuli induced EMT in BxPC-3 cells, which was consistent with the report by Vogelmann R et al [9]. In addition, qRT-PCR demonstrated that RGC-32 mRNA expression was up-regulated significantly at 48 h of TGF-β1 treatment and dramatically increased by about 6 folds at 72 h of treatment (Figure 2B) and western blot showed that RGC-32 protein expression was up-regulated significantly within 48 h of treatment (Figure 2C). These results above indicated that TGF-β enhanced RGC-32 expression as well as inducing EMT in BxPC-3 cells.

## RGC-32 overexpression induces EMT independently in BxPC-3 cells

**Figure 3:**
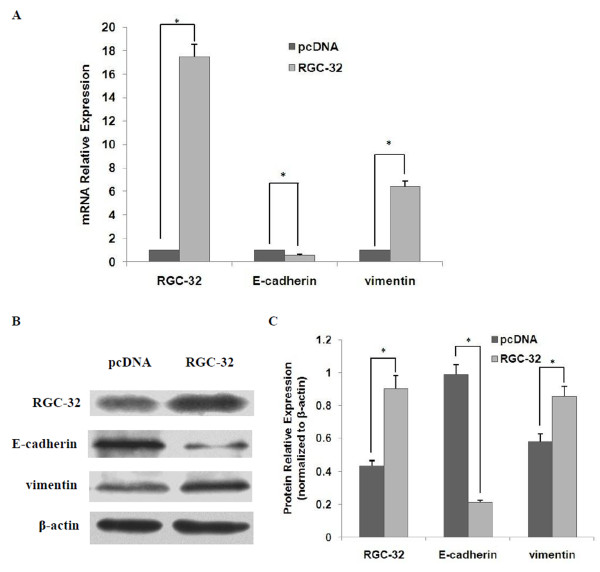
**RGC-32 overexpression promotes EMT of BxPC-3 cells**. BxPC-3 cells were transiently transfected with RGC-32 plasmid (pcDNA3.1/*myc*-His C-RGC32) or empty vector (pcDNA3.1/*myc*-His C). 72 h after transfection, qPCR (A) and western blot (B and C) were performed to examine the expression of RGC-32, E-cadherin and vimentin at mRNA and protein levels respectively. β-actin was used as an internal control. *P < 0.05.

To investigate whether RGC-32 alone could induce EMT in BxPC-3 cells, we transiently transfected RGC-32 plasmid (pcDNA3.1/*myc*-His C-RGC-32) into BxPC-3 cells to overexpress RGC-32. Empty vector (pcDNA3.1/*myc*-His C) was used as a negative control. mRNA expression and protein expression of EMT markers such as E-cadherin and vimentin were detected by qRT-PCR and western blot respectively. As shown in Figure 3, RGC-32 overexpression significantly down-regulated E-cadherin expression and up-regulated vimentin expression at both mRNA and protein levels, indicating that RGC-32 overexpression induced EMT in BxPC-3 cells independently.

## RGC-32 mediates TGF-β-induced EMT in BxPC-3 cells

**Figure 4:**
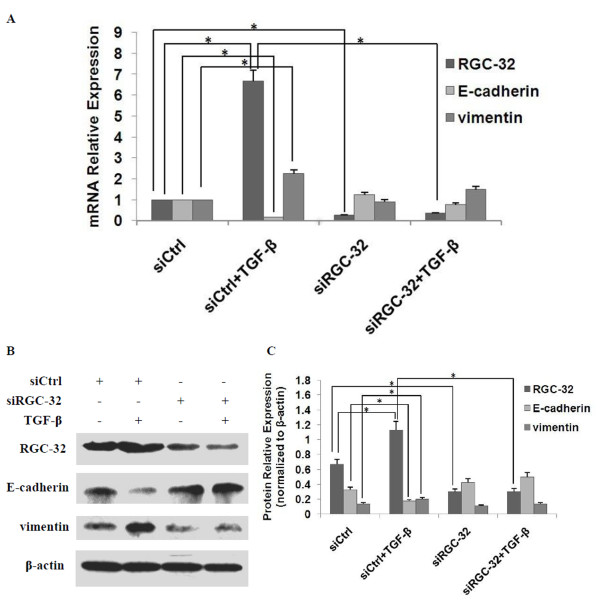
**RGC-32 mediates TGF-β-induced EMT in BxPC-3 cells**. BxPC-3 cells were transfected with RGC-32 siRNA (siRGC-32) or negative control siRNA (siCtrl). 6 h later, cells were starved in serum-free RPMI-1640 for additional 6 h, followed by treatment with or without 10 ng/ml TGF-β1 for 72 h. The mRNA expression and protein expression of RGC-32, E-cadherin and vimentin were examined by qRT-PCR (A) and western blot (B and C) respectively. β-actin was used as an internal control. RGC-32 silencing significantly blocked TGF-β-induced EMT in BxPC-3 cells. *P < 0.05.

We used RNA interference technique to further determine the role of RGC-32 in TGF-β-induced EMT. As shown in Figure 4, compared with the negative control, RGC-32 siRNA transfection significantly attenuated the expression of RGC-32 mRNA and in turn led to the inhibition of RGC-32 protein expression. Meanwhile, TGF-β-induced up-regulation of vimentin and down-regulation of E-cadherin was remarkably blocked by RGC-32 silencing, indicating that RGC-32 played a critical role in TGF-β-induced EMT.

## RGC-32 mediates TGF-β-induced migration of BxPC-3 cells

**Figure 5:**
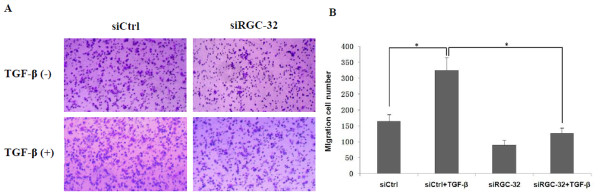
**RGC-32 mediates TGF-β-induced migration of BxPC-3 cells**. BxPC-3 cells were transfected with siRGC-32 or siCtrl and treated with 10 ng/ml TGF-β1 or not as described before. 24 h later, 2 × 10^5 ^cells were loaded into the top chamber of 24-well transwell plates and incubated for another 24 h. The migrated cells were stained with 0.1% crystal violet and the average number per field was quantified under high power (original magnification × 200) of the phase contrast microscope. *P < 0.05.

We used transwell cell migration assay to examine the role of RGC-32 in cell migration of BxPC-3 cells. As shown in Figure 5, TGF-β treatment promoted the migration of BxPC-3 cells while RGC-32 RNA silencing remarkably blocked this effect, implicating that RGC-32 mediated TGF-β-induced migration of BxPC-3 cells.

## Discussion

Recent studies have implicated EMT in cancer progression by showing that epithelial-like tumor cells could switch to a mesenchymal-like phenotype that facilitates motility and invasion [21]. EMT-related molecular pathways have been extensively investigated, and various genes and molecules have been identified as important factors in EMT, of which TGF-β has been most studied and believed to be the major inducer in pancreatic cancer [22]. It has been demonstrated that when TGF-β binds to the TGFβRII, TGFβRI becomes phosphorylated and propagates the signal downstream through phosphorylation and thereby activation of the Smad2 and Smad3 proteins (receptor Smads). The activated receptor Smads form a complex with Smad4 and translocate into the nucleus to regulate the expression of genes involved in EMT [23,24]. Beside Smad-mediated transduction, TGF-β also induces EMT via Smad-independent signaling cascades including PI3K, MAPK, Rho kinase pathways and so on [25]. Our research demonstrated that constant stimuli by TGF-β induced EMT in BxPC-3 cells and the observed changes were proposed to be independent of Smad pathway because Smad4 is homozygous deleted in BxPC-3 cells [26]. The result was consistent with that in Vogelmann R et al's research [9]. However, the downstream effectors of Smad-independent pathways mediating TGF-β-induced EMT remain largely unknown. In particular, it has been reported that mutation or deletion of Smad4 is found in about 50% of pancreatic tumors and is correlated with poor prognosis [27]. Thus, it may be more important to investigate Smad-independent pathways in detail in order to further understand invasion and metastasis of pancreatic cancer.

Recently several studies have shown that RGC-32 plays an important role in EMT. Fengmin Li et al [12] reported that RGC-32, regulated by both Smad and RhoA, participated in TGF-β-induced smooth muscle differentiation from neural crest cells and Wen-Yan Huang et al [28] showed that RGC-32, acting downstream of Smad, mediated TGF-β-induced EMT of human proximal tubular cells (HPTCs). However, as far as we know, there have been no reports about the role of RGC-32 in pancreatic cancer. In this study, by means of immunohistochemical staining, we found for the first time that the expression of RGC-32 was up-regulated in pancreatic cancer and was correlated with lymph node metastasis and TNM staging, which suggested that RGC-32 might be a novel tumor metastasis promoting factor for pancreatic cancer.

E-cadherin is an important epithelial marker for the process of EMT, which has been implicated in cell-cell adhesion and maintenance of normal tissue architecture [29]. E-cadherin interacts at a conserved cytoplasmic domain with the cytoskeleton via associated cytoplasmic molecules, α-, β- and γ-catenin [29]. It has been demonstrated by many researches that abnormalities in expression and function of the adhesion complex have been found in pancreatic cancer and were believed to result in loss of cell-cell adhesion and contribute to the invasiness and metastasis of tumor [30,31]. Immunohistochemical analysis in our research showed that abnormal E-cadherin expression rate was higher in pancreatic cancer tissues than that in chronic pancreatitis and normal pancreatic tissues, and was correlated with clinicopathological features such as tumor differentiation, lymph node metastasis and TNM staging. The results were consistent with those in a research of early gastric cancer [32]. Furthermore, we found for the first time that there was a significant and positive correlation between positive expression of RGC-32 and abnormal expression of E-cadherin, which implicating that RGC-32 might promote metastasis by controlling EMT of pancreatic cancer.

In order to clarify whether RGC-32 is involved in EMT and to investigate its upstream regulator in pancreatic cancer, we focused on its role in TGF-β signaling pathway in vitro. TGF-β-induced-EMT model in BxPC-3 cells showed increased expression of RGC-32 at both mRNA and protein levels, indicating that RGC-32 might be involved in TGF-β-induced EMT. In addition, RGC-32 RNA silencing blocked EMT induced by TGF-β in BxPC-3 cells, confirming that RGC-32 mediates TGF-β-induced EMT. Furthermore, overexpression of RGC-32 demonstrated that RGC-32 can induce EMT independently in BxPC-3 cells. Specially, given that Smad4 is homozygous deleted in BxPC-3 cells, we suppose that RGC-32 induces EMT independent of Smad pathway and that RGC-32 may be a critical effector in Smad-independent pathways. It is important to note that our results were not the same as those in neural crest cells and HPTCs in which RGC-32 is a downstream target of Smad pathways, indicating that the activation pathway and effect of RGC-32 between normal development and carcinogenesis may be controlled by different mechanisms. Finally, by means of transwell cell migration assay we further showed that RGC-32 mediated TGF-β-induced cell migration in BxPC-3 cells, implicating that RGC-32 helps to enhance metastatic phenotype in vitro.

## Conclusions

To sum up, an important issue addressed in this study is that RGC-32 might be a novel metastasis promoting factor for pancreatic cancer and it enhances metastatic phenotype by mediating TGF-β-induced EMT independent of Smad pathway in pancreatic cancer cell line BxPC-3. These findings described for the first time the role of RGC-32 in the progression of pancreatic cancer and indicated that RGC-32 might be a new target for inhibiting metastatic dissemination of pancreatic cancer. Further exploration of the concrete mechanism by which RGC-32 induces EMT is needed to fully understand its role in the process of EMT and metastasis of pancreatic cancer.

## Abbreviations

RGC-32: Response gene to complement-32; TGF-β: Transforming growth factor-β; EMT: Epithelial-mesenchymal transition; PI3K: Phosphoinositide 3 kinase; MAPK: Mitogen-activated protein kinase.

## Competing interests

The authors declare that they have no competing interests.

## Authors' contributions

QZ and LZ designed the experiments. LZ performed most of the experiments and drafted the manuscript. HQ carried out the immunohistochemistry. PYL helped in constructing RGC-32 plasmid. SNX and DML participated in western blot. LZ, HFP and HZZ participated in statistical analysis and interpretation of data. All the authors read and approved the final manuscript.
